# Lipid Perfluorohexane Nanoemulsion Hybrid for MRI-Guided High-Intensity Focused Ultrasound Therapy of Tumors

**DOI:** 10.3389/fbioe.2022.846446

**Published:** 2022-03-31

**Authors:** Sitong Liu, Xiuqi Hou, Wenjian Zhu, Fang Zhang, Weiling Chen, Binjian Yang, Xin Luo, Dalin Wu, Zhong Cao

**Affiliations:** ^1^ School of Biomedical Engineering, Sun Yat-sen University, Guangzhou, China; ^2^ Sun Yat-Sen Memorial Hospital, Sun Yat-Sen University, Guangzhou, China; ^3^ Department of Obstetrics and Gynecology, the First Clinical Medical College of Jinan University, Guangzhou, China; ^4^ Sinopharm Tongmei General Hosptial, Datong, China

**Keywords:** magnetic resonance imaging, superparamagnetic iron oxides, nanoemulsion, perfluorohexane, high-intensity focused ultrasound

## Abstract

Magnetic resonance imaging-guided high-intensity focused ultrasound (MRI-guided HIFU) is a non-invasive strategy of diagnosis and treatment that is applicable in tumor ablation. Here, we prepared a multifunctional nanotheranostic agent (SSPN) by loading perfluorohexane (PFH) and superparamagnetic iron oxides (SPIOs) in silica lipid for MRI-guided HIFU ablation of tumors. PFH was introduced to improve the ablation effect of HIFU and the ultrasound (US) contrast performance. Due to its liquid-to-gas transition characteristic, it is sensitive to temperature. SPIOs were used as an MRI contrast agent. Silica lipid was selected because it is a more stable carrier material compared with normal lipid. Previous studies have shown that SSPNs have good biocompatibility, stability, imaging, and therapeutic effects. Therefore, this system is expected to develop an important therapeutic agent for MRI-guided HIFU therapy against tumors.

## Introduction

High-intensity focused ultrasound (HIFU) is a non-invasive therapy that has broad applications in the treatment of various diseases, especially cancers (Bi et al., 2019). HIFU is able to target deep tumors *in vivo* and cause irreversible death of cancerous cells through rapid heating, while ensuring the safety of skin and the surrounding tissues of the tumor (Dabbagh et al., 2015). However, the application of HIFU is limited because its resolution and efficacy do not meet clinical needs.

Recently, various multifunctional HIFU synergistic agents based on organic microbubbles have been successfully developed. The new agents are capable of enlarging the lesion site, thus improving therapeutic effects (Cheng et al., 2019). In addition, some agents can also monitor the treatment process through medical imaging technologies for more precise therapy (VanOsdol et al., 2017). However, the organic microbubbles cannot penetrate the endothelial space due to their short retention time in blood vessels and large particle size (micron level) (Yang et al., 2015). Fortunately, this problem can be solved with the development of liposome-based synergists (Feng et al., 2017). Liposome-based synergists can accumulate in the tumor site (through the EPR effect), enhance ultrasound imaging, and promote HIFU ablation effect as a result of having internal substances such as fluorocarbon with liquid-to-gas phase transition characteristic (Chen et al., 2015; Cui et al., 2019).

Several studies have been done in this regard. A stable ultrasound-imageable liposome was developed by Danny Maples et al. (2015). It was co-loaded with doxorubicin (DOX) and perfluoropentane (PFP). This agent showed great potential for *in vivo* image-guided drug delivery and HIFU therapy against tumors. Nevertheless, the poor thermal stability of these liposomes limited their wider application. Wang et al. (2012) described perfluorohexane-encapsulated mesoporous silica nanocapsules. The nanocapsules possess excellent stability but the biocompatibility needs to be further improved. Therefore, the development of safe and stable agents that integrate diagnosis and treatment is still the focus (and a problem) of current research.

Magnetic resonance imaging (MRI) is widely used in clinical diagnosis because, without using radiation, it could provide high-resolution 3D tomographic images and excellent soft-tissue contrast (Zhu et al., 2017; Cheng et al., 2019). Integration of MRI and HIFU forms a non-invasive diagnosis and treatment system for accurate tumor ablation. Recently, the rapid development of this technology has made it widely accepted in the clinical treatment of both malignant and benign tumors (Napoli et al., 2013). Thence, exploring new agents for MRI-guided HIFU has attracted widespread attention.

**FIGURE 1:**
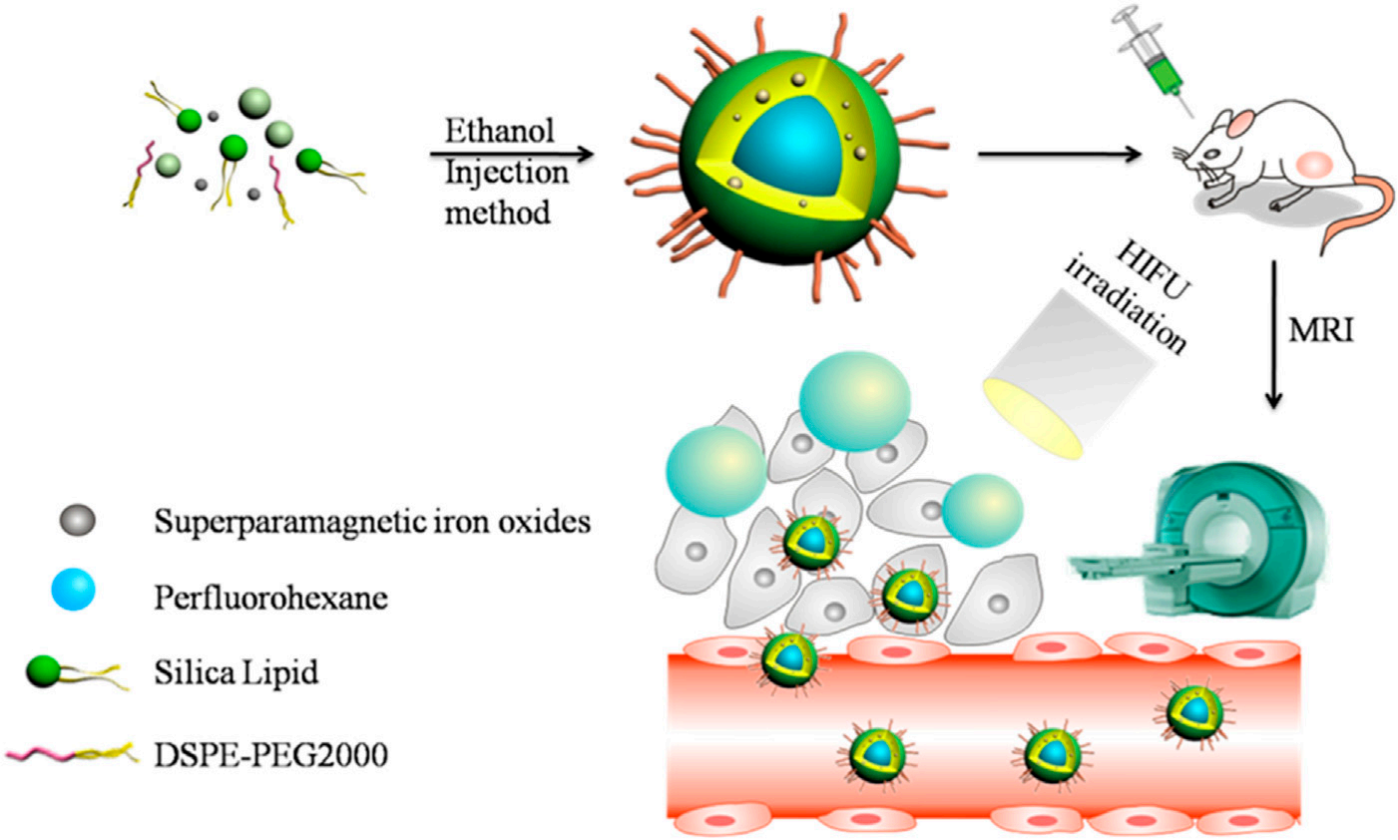
Schematic illustration of SSPN and “liquid-to-gas” phase transition behavior.

This study explored a multifunctional agent with PFH and superparamagnetic iron oxides (SPIOs) loaded in silica lipid (SSPN) (Figure 1). The nano-size nanoemulsion allows it to reach the tumor site through blood circulation and remain at the site through the EPR effect. The silica lipid forms a layer of inorganic polysiloxane network structure on the surface of the nanoemulsion, making SSPN more stable than liposomes; in addition, it has better biocompatibility than silica nanoparticles. Upon HIFU irradiation, the internal PFH of SSPN undergoes transformation from liquid to gas. Therefore, it promotes ultrasound imaging and HIFU ablation effect at the same time. In addition, superparamagnetic iron oxide (effective T2 contrast agent) improves the MRI contrast capability of SSPN.

## Materials and Methods

### Materials

Ethanol, HCl (36%–38%), and NaCl were obtained from Guang Zhou Chemical Reagent Factory (China). From Avanti Polar Lipids Inc. (Birmingham, AL, United States), 1,2-distearoyl-sn-glycero-3-phosphoethanolamine-N-[methoxy (polyethylene glycol)-2000] (DSPE-PEG2000) was purchased for this study. Perfluorohexane, min. 96% was obtained from Strem Chemicals, Inc. (Newburyport, MA, United States). Methylthiazol tetrazolium (MTT) was bought from Sigma–Aldrich Chemical Co. (St. Louis, MO, United States). SPIOs were purchased from Agilent (United States). N-[N-(3-triethoxysilylpropyl) succinamoyl] dihxadecylamine (silica lipid) was prepared through a reported previously procedure (Katagiri et al., 2007). Deionized water (18.2 MΩ cm) was used in all experiments. All chemicals used in the study were of analytical grade.

### Preparation of SSPNs

The typical ethanol injection method was used to prepare SSPNs. First, silica lipid (4 mg, 91 mol%) and DSPE-PEG2000 (9 mol%) were dissolved in ethanol (0.2 ml, pH 3) and incubated at 50°C for 4 h to obtain a hydrophilic Si-OH. Then, SPIOs and PFH (2.5%, v/v) aqueous solution were added and probe sonication was performed to obtain SSPNs, with a layer of polysiloxane network structure and PEG on the surface of the nanoemulsion. Finally, the sample was centrifuged (6,000 rpm, 3 min), dispersed in water, and stored at 4°C for later use. Using the same method, SNs (blank silica lipid nanoparticles) and SPNs (PFH-loaded silica lipid nanoparticles) were prepared for the control groups.

### Characterizations of SSPNs

Malvern Zetasizer Nano-ZS90 (Worcestershire, United Kingdom) was used to measure the mean size and zeta potential of SSPNs through dynamic light scattering (DLS) method. Transmission electron microscopy (TEM) (JEM1400, JEOL Co., Japan) was carried out to characterize morphologies of SSPNs. Briefly, the samples were introduced into a carbon-coated grid and staining was done using uranyl acetate solution (1%, w/v) after the removal of water had been done.

Stability of SSPNs was assessed by dispersing 200 μg of freshly prepared samples in phosphate buffer saline (PBS, pH 7.4) containing 10% fetal bovine serum (FBS). Then, this system was placed in a shaker (37°C, 1,000 rpm) and the particle size of the samples was detected at different times. Inductively coupled plasma atomic emission spectrometry (ICP-AES) was used to determine the content of SPIOs in SSPNs. Rigaku D/Max-IIIA x-ray diffractometer with a Cu target (40 kV, 40 mA) was used to obtain the XRD patterns of SPIOs and SSPNs. Magnetization data were measured using MPMS XL-7 Quantum Design SQUID magnetometer with an applied magnetic field set between 2 × 10^4^ Oe to and 2 × 10^4^ Oe at 300 K.

### Polarizing Optical Microscopy Imaging

Polarizing optical microscopy (LEICA DM 2500P) with a hot stage (Linkam) was used to determine the effect of temperature on SSPNs. A 20 μl solution of SSPNs was added on a glass slide and covered with a cover glass. To avoid evaporation of water, the edges of the cover glass were sealed before being placed on the hot stage. Images were obtained before and after heating (at 60 and 65°C) using the polarizing optical microscopy equipped with a 40× focal lens to locate the SSPNs. The same procedure was carried out with SNs and SPNs as the control samples to observe different behaviors during heating.

### *In vitro* US Imaging

Toshiba ultrasound system was used to receive US images and confirm potential of US contrast *in vitro*. SSPNs were transferred into plastic droppers after being diluted with degassed water at different multiples. The Toshiba ultrasound system with linear array transducer was used for imaging of SSPNs in the plastic droppers placed in a sink. The contrast mode was set at a frequency of 3.5 MHz and at a mechanical index of 0.08. Ultrasound images of SSPNs were taken before and after 30 s of heating at 60 and 65°C. ImageJ (National Institutes of Health, United States) was used to measure the average of the gray-scale levels of all pixels within a manual-defined ROI to obtain the gray values of US images.

### *In vitro* MR Imaging

SPIOs and SSPNs with different concentrations of Fe were added in 96-well plate, 200 μl per well. A clinical MRI instrument (Philips Intera 1.5 T) was used to scan T2-weighted MR images. The MRI instrument used a circular coil (11 cm) and a single-section mixed inversion-recovery spin-echo sequence (stepped echo time of 20–160 ms for eight steps, echo spacing of 20 ms, FOV of 70 mm, a matrix of 256 × 256, and a section thickness of 1 mm). The T2 relaxation value was calculated from the linear of 1/T2 relaxation time (s^−1^) versus the Fe concentration (mM) to compare the contrast effects of SPIOs and SSPNs. The MRI signal intensity of tumors was measured by using ImageJ software (NIH, Bethesda, MD, United States) on T2WI at each time point. Tumor signal intensity was measured with irregular region of interest (ROI) on T2WI.

### *Ex vivo* HIFU Ablation

A HIFU therapy system (PRO, China) was used to perform HIFU therapy experiment *ex vivo*. Pig livers were degassed by an ultrasonic cleaner. They were later placed on the bottom of a water tank filled with degassed water before the HIFU irradiation was performed. The HIFU irradiation (210 W, 6 s) was applied immediately after injection of the pig livers with experiment samples (2 ml, 2.5 mg ml^−1^) including normal saline (NS), SNs, and SSPNs. Recording of B-mode imaging was carried out every time before and after HIFU irradiation was performed. The mode was set at a frequency of 3.5 MHz and at a mechanical index of 0.08. The related ablation volumes were calculated using the following formula:

$$V=\pi \times L\times W^{2}/6$$

where *L* and *W* represent the maximum length (mm) and maximum width (mm) of the coagulated livers, respectively.

All experiments were performed in triplicate.

### Cell Viability

Application of nanoparticles is affected by cytotoxicity. HeLa cells were used to detect cytotoxicity of SPNs and SSPNs. The cells were seeded into the 96-well plate at a density of 2,500 cells per well in 100 μl of Dulbecco’s Modified Eagle Medium (DMEM). After incubation for 12 h, the medium (DMEM) was replaced with a fresh medium (100 μl) containing different concentrations of the samples. After a 48-h incubation period, MTT assay was performed to quantify the cell viability.

### *In vivo* MR Imaging

*In vivo* experiments used BALB/c mice (5 weeks, 18–22 g) as the animal models. Ethical approval of this study was given by the institutional animal care and use committee of the Sun Yat-sen University. Murine CT26 cells were used to establish xenografts in mice. The right back fur of mice was removed with an electric razor, and CT26 cells at a density of 1 × 10^6^ and in 100 μl of PBS were subcutaneously injected into the right back of the mice. The mice were used when their tumor volumes approached 60–80 mm^3^ as measured by a caliper:

$$V=0.5\times L\times W^{2}$$

where *L* and *W* represent the length and the width of tumors, respectively.

*In vivo* MR imaging was evaluated using a clinical MRI instrument (Siemens 3T). Intraperitoneal injection of 30 μl of pentobarbital sodium (3%, w/w) was used to anesthetize the mice. Each mouse was injected with 100 μl of SSPNs (10 mg ml^−1^) for tumor imaging. The MR images were taken at different times of the experiment period. Parameters of T2-weighted MRI were fixed as follows: TR/TE, 200/14.7 ms; flip angle, 25°; FOV, 80 mm; matrix, 256 × 256; and slice thickness, 1 mm.

### *In vivo* HIFU Ablation

For *in vivo* HIFU experiment, BALB/c mice bearing CT26 murine colon cancer tumors were randomly divided into 3 groups (NS, SNs, and SSPNs). The animals were anesthetized using intraperitoneally injected pentobarbital sodium (30 μl, 3% w/w). Anesthetized mice were placed under a degassed water tank with a hole in the bottom. All the mice were injected with the samples (100 μl, 10 mg ml^−1^) through the tail vein. Thirty minutes later, HIFU irradiation (210 W, 6 s) was conducted. The B-mode imaging was recorded before and after HIFU irradiation. The mode was set at a frequency of 3.5 MHz and at a mechanical index of 0.08. The mice were sacrificed 24 h after irradiation and developed tumors were collected for analysis.

The volumes of related ablation tumors were calculated using the following formula:

$$V=\pi \times L\times W^{2}/6$$

where *L* and *W* are the maximum length (mm) and width (mm) of the tumors, respectively.

All experiments were performed in triplicate. Then, the tissue sections were stained using hematoxylin-eosin (H&E) stain following the standard protocol.

### Statistical Analysis

The group differences are indicated as mean ± standard deviation (SD). Differences between each group were assessed by Student’s *t*-test, while *p < 0.05 was regarded as statistically significant, **p < 0.01 was highly significant, and ***p < 0.001 was strongly significant.

## Results and Discussion

### Characterizations of SSPNs

**FIGURE 2:**
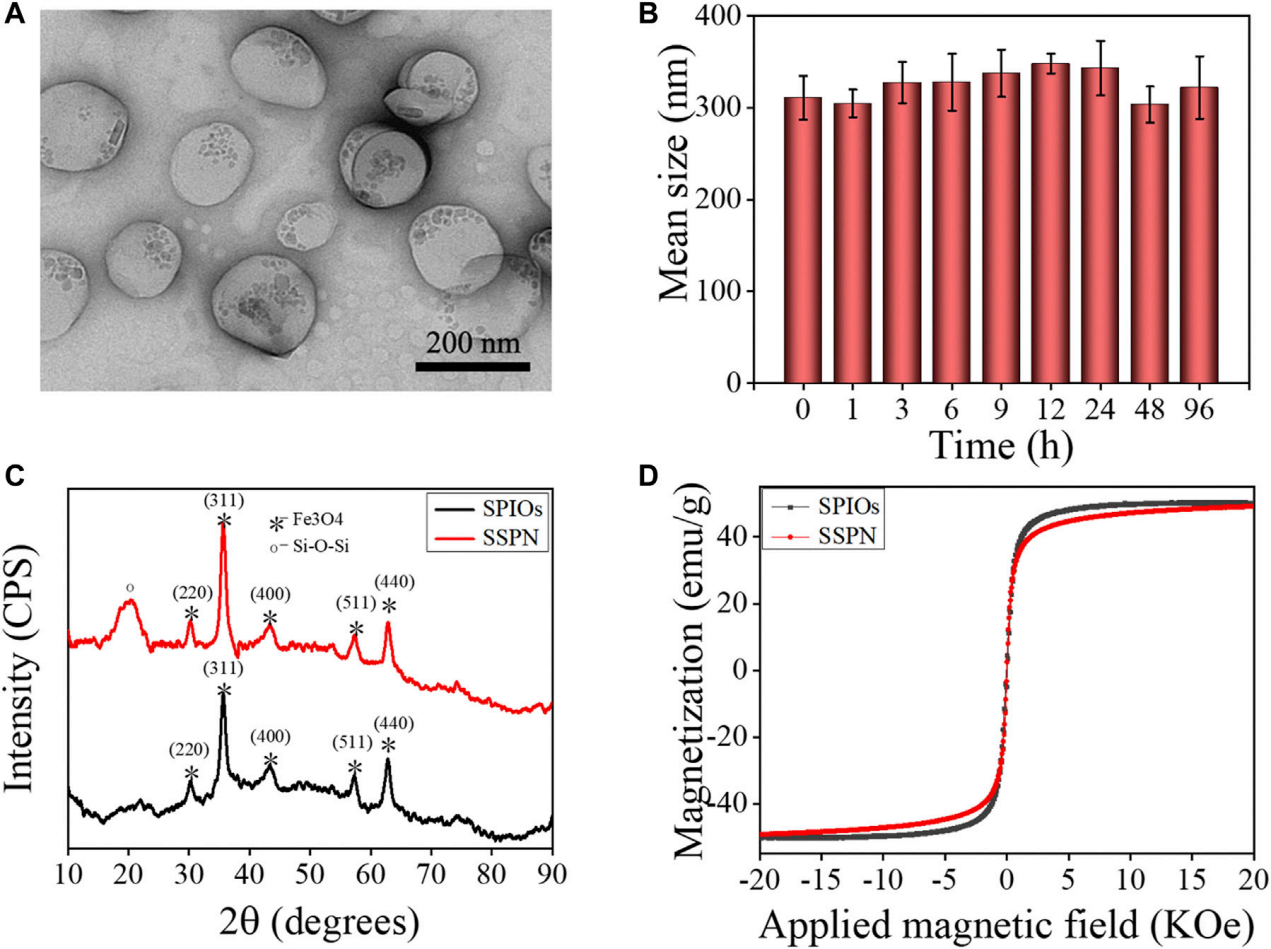
Characterizations of SSPNs. **(A)** TEM image. Scale bar = 200 nm. **(B)** The stability of SSPNs in PBS containing 10% FBS in 96 h. **(C)** XRD spectrums of SPIOs and SSPNs. **(D)** Magnetization curves of SPIOs and SSPNs.

Related characterizations were performed to prove the success of SSPN preparation and evaluate their properties (Figure 2). The SSPNs showed spherical structures with uniform particle size and good dispersion. The small particles on the nanoemulsion showed that SPIOs were loaded successfully (Figure 2A). The mean size of SSPNs analyzed by DLS was 289 nm, which was consistent with the TEM result. The zeta potential of SSPNs decreased from −31.6 ± 3.8 mV to −15.6 ± 2.6 mV after modification of PEG. The decrease might be attributed to the formation of a hydration layer on the surface of the nanoemulsion to shield certain charges. The decreased surface potential was important for stability improvement. As shown in Figure 2B, there was no significant change in size after being dispersed in PBS (10% FBS) for a long period up to 8 days, indicating that SSPNs possessed excellent stability in physiological environment.

The XRD spectrums of SPIOs and SSPNs are displayed in Figure 2C. SPIOs showed characteristic peaks at (220), (311), (400), (511), and (440), which were comparable with the standard tetragonal phase (JCPDS Card No. 75-1,594). Similar wide-angle XRD peaks appeared in SSPNs and SPIOs, suggesting that SPIOs were successfully loaded in SSPNs. The peak at 23 revealed the siloxane structure. According to the ICP-AES, the Fe content was 6.7 wt%. The superparamagnetic behaviors with zero coercivity and remanence were proved by the saturation magnetization curves of both SPIOs and SSPNs (Figure 2D). The saturation magnetizations were 58.4 Fe emu g^−1^ for SPIOs and 57.8 Fe emu g^−1^ for SSPNs. There was no obvious loss in magnetization per Fe unit. Therefore, SSPNs have potential as a contrast agent for MRI.

### Temperature-Responsive Phase Transformation of SSPNs

**FIGURE 3:**
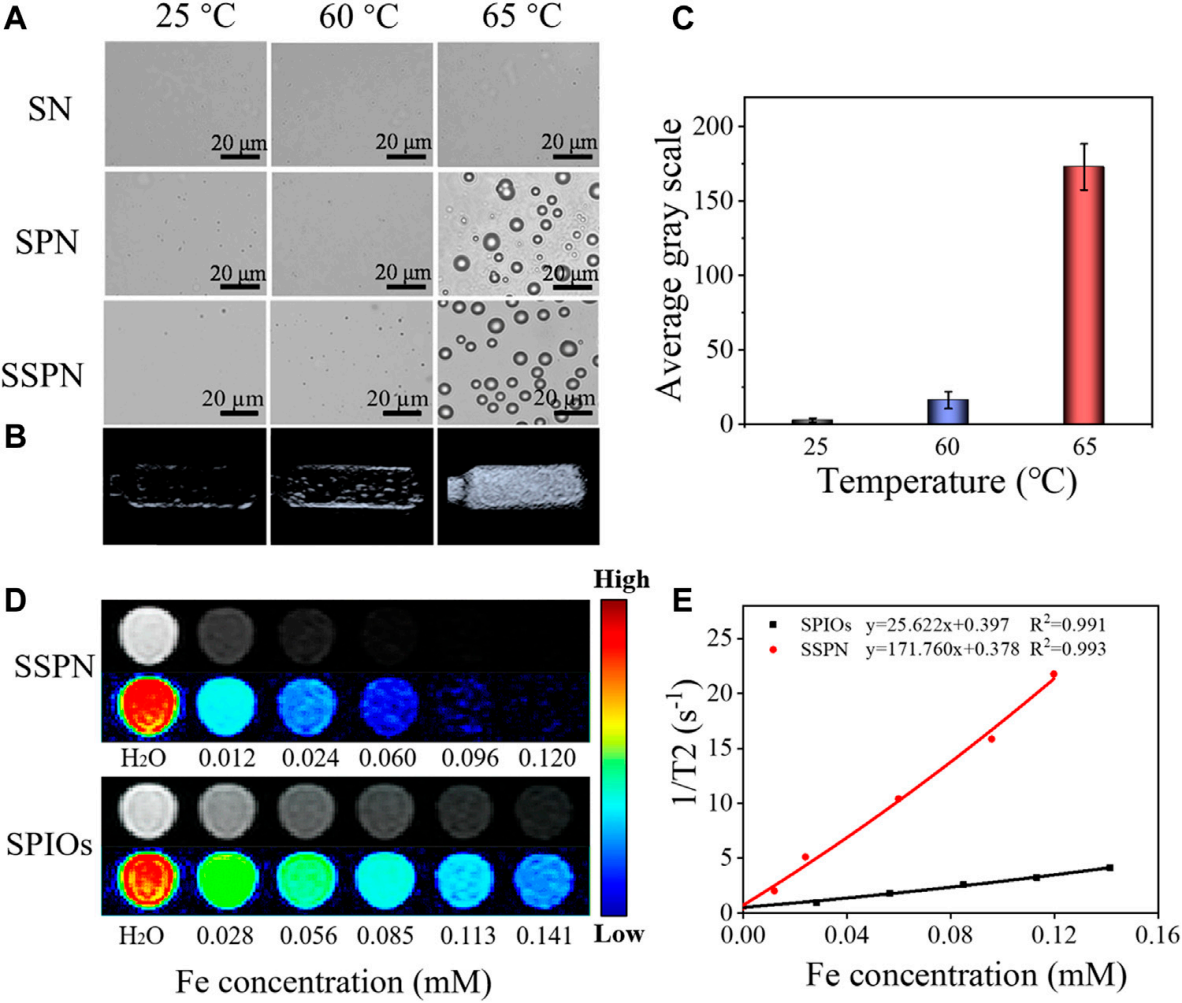
**(A)** Polarizing microscope images of SNs, SPNs, and SSPNs before and after being heated from 25°C to 65°C (scale bar = 20 μm). **(B)** *In vitro* US images of SSPNs at 25°C, 60°C, and 65°C and **(C)** average gray values of SSPNs (*n* = 3). **(D)** T2-weighted MR images of SSPNs and SPIOs at different Fe concentrations using a 1.5-T MRI scanner. **(E)** T2 relaxation rates of SSPNs and SPIOs corresponding to **(D)**.

The basis for the use of SSPNs in HIFU ablation and ultrasound imaging was the temperature-responsive phase transition property of PFH. The behaviors of SNs, SPNs, and SSPNs heated from 25 to 65°C are observed in Figure 3A. At 25°C, all the three samples had no significant change. When temperature was increased to 60°C, no significant change was observed in the SN and SPN group, but some bubbles were generated in the SSPN group, demonstrating that a small part of the PFH underwent a transition from liquid phase to gas phase. The bubble formation was evidently observed in the SPN and SSPN group after the temperature was increased to 65°C, suggesting that most of the PFH were converted into gas. In contrast, the SN group maintained their original state throughout experiment period. As was known to us, the boiling point of PFH was usually between 58 and 60°C, but here, the boiling point of PFH was increased by the Laplace pressure difference between the inside and outside of the silica lipid shell to a certain extent (Yang et al., 2018). The inconsistent behaviors of SPNs and SSPNs could be due to the SPIOs loaded between the double-layer membranes, which affected the pressure difference. Liquid-gas transition of PFH in SSPNs would contribute to ultrasound contrast and HIFU ablation.

### *In vitro* US Imaging

Furthermore, SSPNs heated to different temperatures were imaged by US correspondingly, and the ultrasound intensity values were quantitatively analyzed. According to Figures 3B,C, US imaging effect could be improved by the cavitation induced by bubbles, and the bubble production was directly proportional to the ultrasonic effect. The ultrasound signals were weak at a temperature of 25°C because of the lack of bubbles, while few bubbles resulted into a slight signal observed at 60°C, whereas the large number of bubbles generated at 65°C caused a sharp increase, and the average gray scale was increased 8 times compared with the average gray scale at 60°C. These results show that SSPNs have the potential to enhance ultrasound contrast.

### *In vitro* MR Imaging

The loaded SPIOs enabled SSPNs as T2-weighted MR imaging contrast. In order to compare the effects of SPIOs before and after being loaded in SSPNs, *in vitro* MR imaging was performed. The MR images of the two samples gradually became darker with the increase of Fe concentration. It was worth noting that SSPNs had better contrast effect compared with SPIOs (Figure 3D). Furthermore, the r2 relaxation rate of SSPNs was 171.76 mM Fe^−1^ s^−1^, which was much higher than the r2 relaxation rate of SPIOs (25.62 mM Fe^−1^ s^−1^) (Figure 3E). This could be attributed to the aggregation of SPIOs in the nanoemulsion. These results evidently suggested that SSPNs could be used as an excellent MRI contrast agent due to the introduction of SPIOs.

### *Ex vivo* HIFU Ablation

**FIGURE 4:**
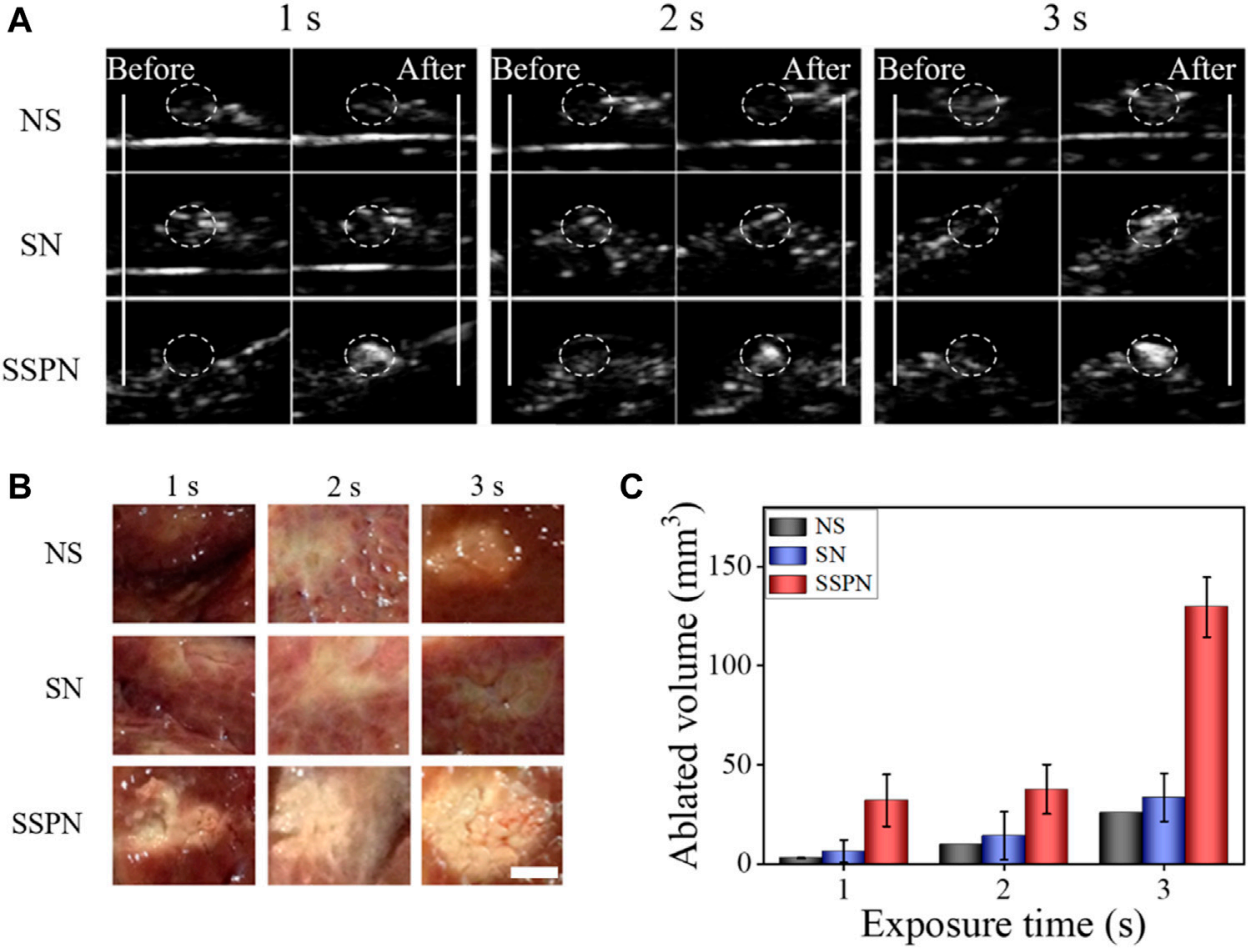
**(A)** B-mode ultrasound images of NS, SN, and SSPN before and after HIFU irradiation on pig livers at 270 W for 1, 2, and 3 s. **(B)** Digital photos of ablated degassed pig livers at 270 W cm^−2^ for 1, 2, and 3 s (scale bar = 5 mm). **(C)** The corresponding necrotic volumes after HIFU irradiation (*n* = 3).

Degassed pig livers were used to carry out the *ex vivo* HIFU experiments. In order to evaluate the effect of HIFU therapy with different samples, B-mode US imaging was used and ablation volumes were calculated (Figure 4). The necrotic volumes and gray-scale areas of the SSPN group (1 s: 30.0 ± 13.1 mm^3^; 2 s: 37.6 ± 12.5 mm^3^; 3 s: 129.5 ± 35.1 mm^3^) were larger than those of NSs (1 s: 2.9 ± 0.3 mm^3^; 2 s: 9.8 ± 10.3 mm^3^; 3 s: 25.8 ± 2.1 mm^3^) and SNs [1 s: 6.2 ± 5.7 mm^3^; 2 s: 14.1 ± 12.1 mm (Cheng et al., 2019); 3 s: 33.5 ± 12.1 mm^3^] after HIFU exposure. The rapid increase in temperature and a liquid-to-gas transition of PFH in SSPNs enhanced acoustic signals and ablation effect significantly.

### *In vitro* Cytotoxicity

**FIGURE 5:**
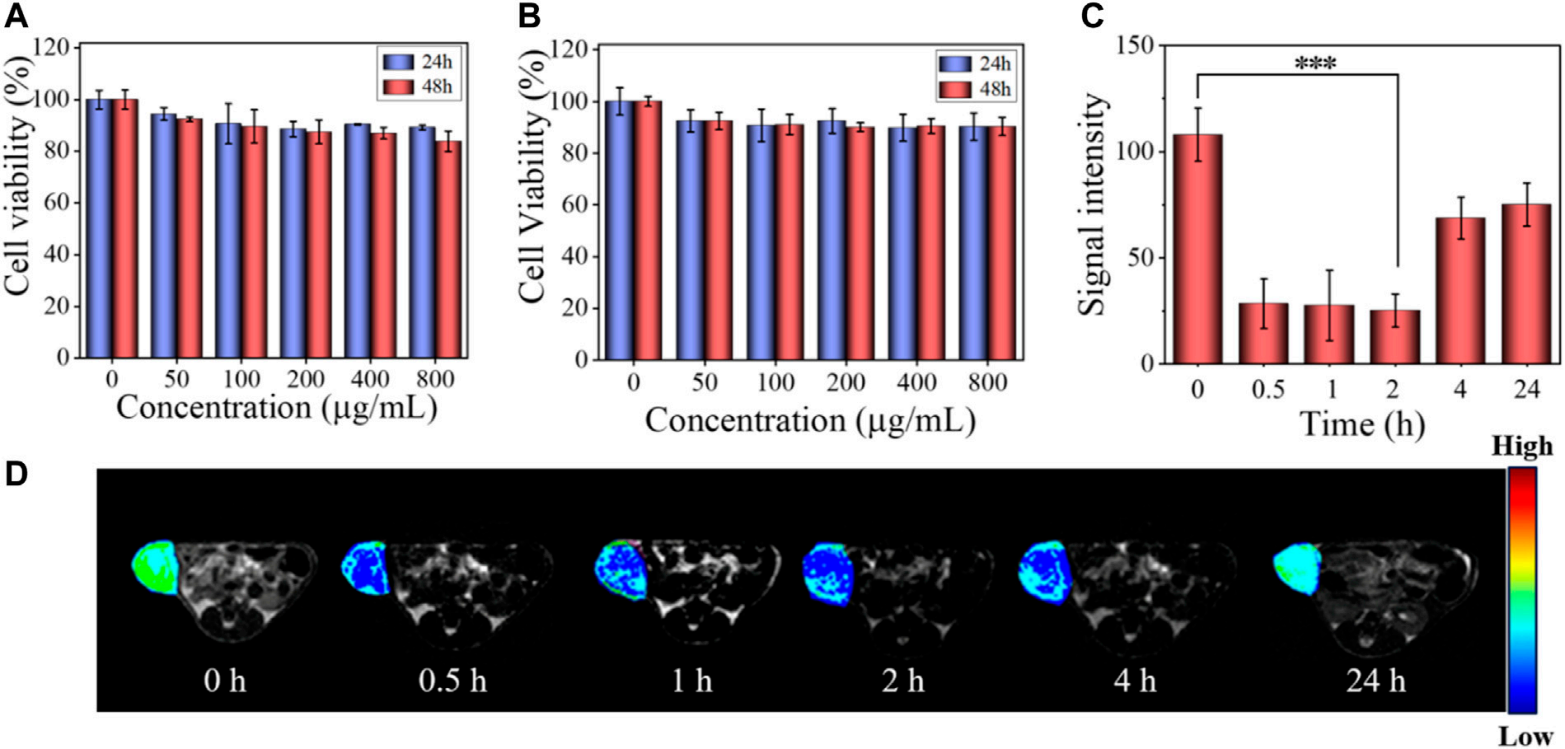
**(A)** Viability of HeLa cells after incubating with SPNs for 24 and 48 h. The data are given as mean ± SD (*n* = 5). **(B)** Viability of HeLa cells after incubating with SSPNs for 24 and 48 h (*n* = 5). **(C)** Signal intensity of *in vivo* MR imaging corresponding to **(D)** (*n* = 3,***p < 0.001, **p < 0.01, *p < 0.05). **(D)** *In vivo* MR images of tumor after intravenous injection of SSPNs (100 μl, 10 mg ml^−1^) at different times.

Cytotoxicity was an important factor to determine whether a material could be used in organisms. The cell viabilities after treatment with SPNs and SSPNs are shown in Figures 5A,B, respectively. All of them were more than 90%, indicating good biocompatibility of SPNs and SSPNs.

### *In vivo* MR Imaging

*In vivo* MR imaging of tumor-bearing mice was performed to explore the T2-weighted MR imaging contrast effect of SSPNs (Figures 5C,D). Obviously, there was a significant decrease in MRI signal strength from 0 to 0.5 h, which could be explained by the fact that SSPNs accumulated at the tumor site through the EPR effect during blood circulation. In the first 2 h of observation, the MRI signal intensity decreased by about 70%, and then gradually recovered after 4 h, which might be due to the removal of SSPNs with blood circulation (Kato et al., 2015). Accordingly, SSPNs could be used for MRI diagnosis of tumor.

### *In vivo* HIFU Ablation

**FIGURE 6:**
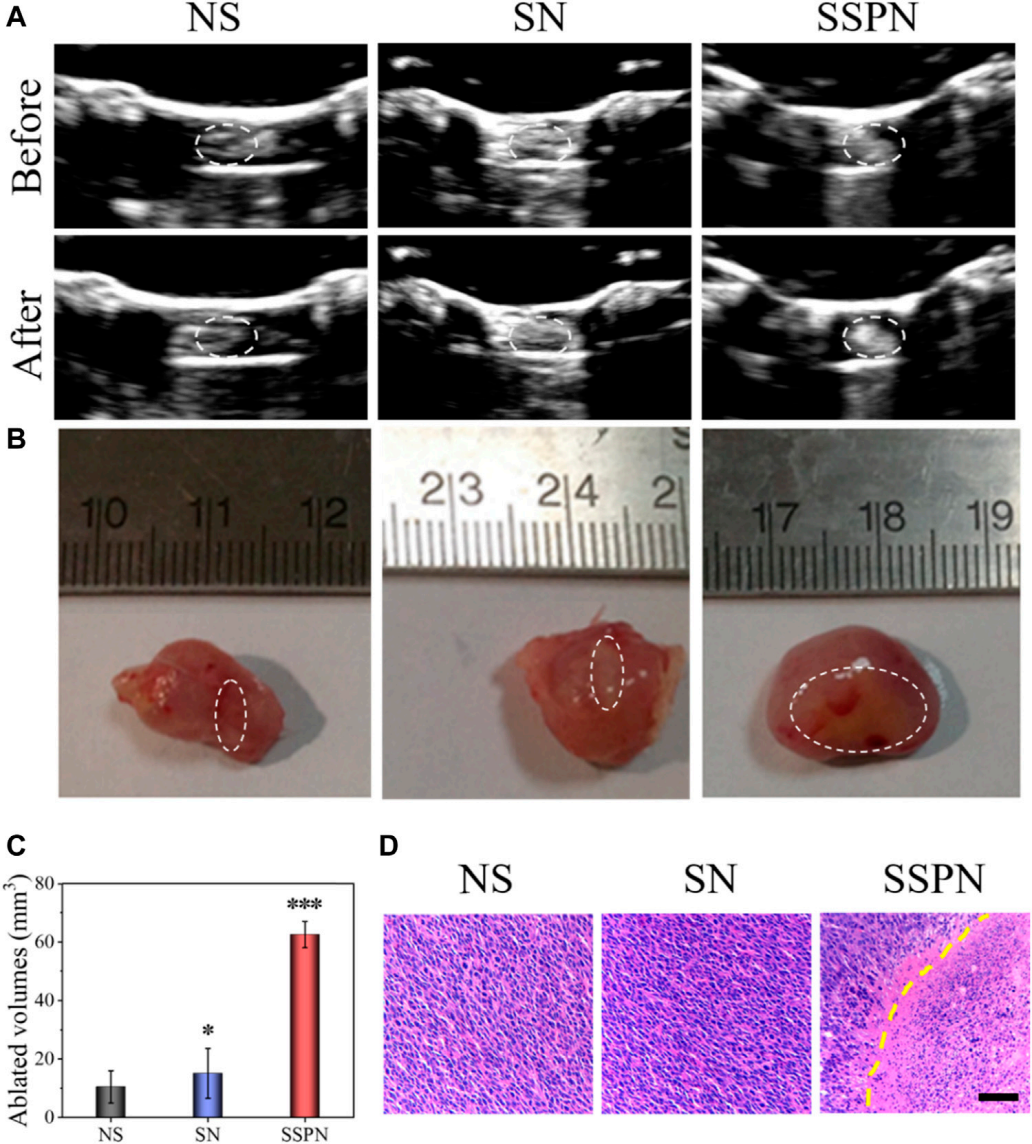
**(A)** B-mode ultrasound images of NS, SNs, and SSPNs before and after HIFU irradiation. **(B)** Digital photos of ablated tumors. **(C)** Quantification of the corresponding necrotic volume after HIFU irradiation (*n* = 3,***p < 0.001, **p < 0.01, *p < 0.05). **(D)** H&E staining of tumor sections after HIFU ablation; images were acquired at 200× magnification (scale bar = 50 µm).

*In vivo* HIFU therapy was performed to further confirm the imaging and treatment potential of SSPNs. Due to the absence of PFH, the NS and SN control groups showed no acoustic signal change before and after HIFU irradiation (Figure 6A). In contrast, the signal of the SSPN group increased obviously because of the cavitation, acoustic streaming, and shear stresses. The necrotic volumes and gray-scale areas of the SSPN group (62.5 ± 14.5 mm^3^) were 5.9 and 4.1 times bigger than that in the NS group (10.5 ± 5.5 mm^3^) and SN group (15.1 ± 8.5 mm^3^), respectively (Figures 6B,C). Cavitation is one of the effects by which the HIFU induces mechanical tissue injury. The liquid-to-gas transition of PFH enhances the cavitation effect, resulting in violent oscillations and rapid growth of the bubble during the rarefaction phase, which eventually leads to its violent collapse and destruction. Bubbles burst explosively, leading to the occurrence of tissue necrosis, and this would cause a strong mechanical injury to the tissue and then form a cavity within the ablated tissue (Chung et al., 2012). Pathological examinations (H& E staining) were used to examine the damage of tumors after treatment (Figure 6D). The HIFU-irradiated cells in the SSPN group were damaged, and a clear boundary between the necrotic region and non-necrotic region was observed, while there was absence of cell necrosis in the NS and SN groups. Therefore, SSPNs were expected to be used as an effective therapeutic agent for HIFU ablation of tumors.

## Conclusion

The results of this study evidently showed the prepared SSPNs with uniform structure, excellent stability, and biocompatibility. The SSPNs could accumulate at the tumor through the EPR effect, and furthermore, since SPIOs and PFH were co-loaded in the nanoemulsion, SSPNs could be used not only for US and T2-weighted MR imaging but also as a therapeutic agent for HIFU therapy. SSPNs provided a possible strategy for cancer diagnosis and treatment with high precision, and have a potential to be developed as a promising agent for MRI-guided HIFU therapy.

## Data Availability

The original contributions presented in the study are included in the article/Supplementary Materials, further inquiries can be directed to the corresponding authors.
